# Peripheral nerve‐derived Sema3A promotes osteogenic differentiation of mesenchymal stem cells through the Wnt/β‐catenin/Nrp1 positive feedback loop

**DOI:** 10.1111/jcmm.18201

**Published:** 2024-04-03

**Authors:** Jingcun Shi, Bingqing Zhang, Ziqian Wu, Yuhan Zhang, Anand Gupta, Xudong Wang, Jieyu Wang, Lisha Pan, Meng Xiao, Shijian Zhang, Lei Wang

**Affiliations:** ^1^ Department of Oral and Maxillofacial Surgery – Head & Neck Oncology Shanghai Ninth People's Hospital, Shanghai Jiao Tong University School of Medicine Shanghai China; ^2^ College of Stomatology Shanghai Jiao Tong University Shanghai China; ^3^ National Center for Stomatology National Clinical Research Center for Oral Diseases; Shanghai Key Laboratory of Stomatology Shanghai China; ^4^ Department of Dentistry, Oral Health Centre Government Medical College Hospital Chandigarh India; ^5^ Department of Stomatology Shanghai East Hospital, School of Medicine, Tongji University Shanghai China; ^6^ Department of Prosthodontics Shanghai Ninth People's Hospital, Shanghai Jiao Tong University School of Medicine Shanghai China; ^7^ Department of Stomatology Fengcheng Hospital Shanghai China

**Keywords:** bone homeostasis, bone remodelling, dorsal root ganglion, mesenchymal stem cells, Neuropilin 1, osteogenic differentiation, osteoporosis, peripheral nerve, Semaphorin 3A, Wnt signalling

## Abstract

Sensory nerves play a crucial role in maintaining bone homeostasis by releasing Semaphorin 3A (Sema3A). However, the specific mechanism of Sema3A in regulation of bone marrow mesenchymal stem cells (BMMSCs) during bone remodelling remains unclear. The tibial denervation model was used and the denervated tibia exhibited significantly lower mass as compared to sham operated bones. In vitro, BMMSCs cocultured with dorsal root ganglion cells (DRGs) or stimulated by Sema3A could promote osteogenic differentiation through the Wnt/β‐catenin/Nrp1 positive feedback loop, and the enhancement of osteogenic activity could be inhibited by SM345431 (Sema3A‐specific inhibitor). In addition, Sema3A‐stimulated BMMSCs or intravenous injection of Sema3A could promote new bone formation in vivo. To sum up, the coregulation of bone remodelling is due to the ageing of BMMSCs and increased osteoclast activity. Furthermore, the sensory neurotransmitter Sema3A promotes osteogenic differentiation of BMMSCs via Wnt/β‐catenin/Nrp1 positive feedback loop, thus promoting osteogenesis in vivo and in vitro.

## 1 INTRODUCTION

Patients with spinal cord injury present significantly decrease in bone mineral density, indicating the potential influence of nerves on bone remodelling.^1^ In fact, nerve fibres are often present in bone trabeculae, periosteum and callus formed around the fracture end.^2^ These observations suggest that nerves play a critical role in regulating bone homeostasis by secreting a variety of important factors such as neurotransmitters, neuropeptides, neuronutrients and neuronal guiding factors.^3^ Notably, recent studies have shown evidence of BI (blood‐innervated) homeostasis between the bone and nervous system, which is marked by the expression of these factors and their receptors in various cell lines.^4^ Our previous studies have revealed that growth factors or neurotransmitters promote nerve repair and regulate bone remodelling. Sensory nerve injuries lead to decreased quality of new bone formation.^5^, ^6^, ^7^, ^8^ Furthermore, retrospective clinical studies have demonstrated that the blood‐innervated double system can effectively prevents osteoporosis and improves the initial stability of the implant bone.^9^ Taken together, these findings suggest that sensory nerves play a critical role in bone metabolism, physiological remodelling, fracture healing and other processes.

Sensory nerves regulate bone nutrition and metabolism primarily through the secretion of neuropeptides that act as endocrine neurotransmitters and neuromodulators.^3^, ^4^ These endocrine neurotransmitters bind to osteoblasts (OBs), osteoclasts (OCs) and bone marrow mesenchymal stem cells (BMMSCs) to regulate bone formation and resorption.^10^ Sensory neurons secrete NGF, Calcitonin gene‐related peptide (CGRP), Substance P (SP), Semaphorin 3A (Sema3A) and other sensory neurotransmitters to promote osteogenesis.^11^, ^12^ Sema3A, a membrane‐related secreted protein in the central nervous system, is involved in guiding axon growth, neuron migration, angiogenesis and tumorigenesis.^13^, ^14^ Studies have shown that mice lacking neuro‐specific Sema3A exhibit a low bone mass phenotype, while OBs‐derived Sema3A deficiency does not affect bone mass, indicating the positive role of sensory nerve‐derived Sema3A in bone homeostasis.^15^ As a result, the bone protective effect of Sema3A has made it a research hotspot.^16^

Sema3A, an axon‐directing factor, regulates bone homeostasis by promoting OBs activity and inhibiting OCs differentiation.^7^, ^17^, ^18^, ^19^ Sema3A binds to two major receptors, Neuropilin 1 (Nrp1) and Plexin A1 (PlxnA1). The binding of Sema3A induces conformational changes in PlxnA1 as it complements the domain of Nrp1, resulting in the activation of intracellular signals.^20^ The induction of dental pulp stem cells (DPSC) by Sema3A leads to the accumulation of β‐catenin in the nucleus, resulting in reduced expression in the cytoplasm.^21^ Ma et al.^19^ found that wedelolactone could promote the formation of Sema3A‐Nrp1‐Plxna1 complex and activate the classical Wnt/β‐catenin signalling pathway. Wedelolactone treatment increased the nuclear accumulation of β‐catenin, which could be reversed by Sema3A antibody. Interestingly, reducing Nrp1 expression with siRNA resulted in decreased β‐catenin expression.^22^ Sema3A can significantly improve Wnt pathway activity and promote upstream and downstream gene expression, such as wnt3a, wnt10a, and axin2, thus promoting osteogenic differentiation.^17^, ^23^ Overall, these findings highlight the importance of Sema3A in regulating bone homeostasis through the classical Wnt/β‐catenin signalling.

Studies have shown that Sema3A therapy is effective in preventing bone loss in ovariectomized mouse models.^24^ Furthermore, local injection of Sema3A into a rat model of osteoporosis has been found to significantly increase callus size and bone density after bone injury, enhance calcium salt deposition and support functional remodelling of the callus 2 months after fracture.^25^ Additionally, a Sema3A knockout mouse model with sensory nerve defects demonstrated reduced bone regeneration.^15^ These findings indicate that Sema3A has potential to treat bone ageing diseases such as osteoporosis and may also serve as a marker for bone diseases.

The current study aims to investigate the molecular mechanism of Sema3A‐mediated regulation of osteogenic differentiation of BMMSCs, through the WNT/Nrp1 positive feedback loop. The results of this study provide a solid and systematic experimental basis for understanding the neuroregulation of bone remodelling and development of biomimetic tissue‐engineered bone.

## 2 METHODS

### 2.1 Construction of the mouse model of tibial denervation

Six weeks old male C57BL/6 mice were provided by the Animal Experimental Center of Ninth People's Hospital affiliated to Shanghai Jiao Tong University (Shanghai, China). Mice were housed as 6/cage and were maintained in a 12 h light/dark cycle at 25°C and were provided with standard rodent chow as well as water. All animal care and procedures were approved by Shanghai Ninth People's Hospital Ethics Committee (SH9H‐2019‐A334‐1).

In this study, bilateral tibia autogenous control with sciatic nerve dissection on the test side and a sham operation on the control side (where only sciatic nerve isolation was performed without amputation) was performed. A total of 23 mice were included, five mice in 1 week group, six mice in 2 weeks group, seven mice in 4 weeks group and five mice in 8 weeks group. Tibia bones were collected at 1 week, 2 weeks, 4 weeks and 8 weeks after operation and prepared for micro‐CT examination. Tibia collected at 4 weeks were also prepared for histological analysis.

### 2.2 The distribution of neurons in the tibia was detected by glycine silver staining

The tibia was fixed in 4% paraformaldehyde for 48 h at 4°C and decalcified at 10% ethylenediamine tetraacetic acid for 4 weeks until the bone became flexible and could be penetrated by needle. The samples were then paraffin embedded and sliced at a thickness of 5 μm. Dewaxing and rehydrating slices were followed by staining according to the instructions of the glycine silver stain kit (Servicebio, China).

### 2.3 HE staining

The bone was decalcified, paraffin embedded and section dewaxed as previously mentioned. The sections were completely immersed in haematoxylin solution for 3–5 min and then washed under running water. Eosin dyeing followed by neutral gum seal piece was done.

### 2.4 Apoptosis was characterized by TdT‐mediated dUTP nick‐end labeling (Tunel) staining

The bone was decalcified, paraffin embedded and section dewaxed to water as previously mentioned. The Cell Apoptosis Detection Kit (Beyotime, China) was used as per the manufacturer's instructions. Anti‐fluorescence quenching tablet encapsulation was utilized, and under a 400× high‐power fluorescence microscope, three fields were randomly selected in each section to observe the distribution of apoptotic cells.

### 2.5 Immunohistochemical staining

Bone decalcification, embedding, dewaxing and other experimental operations were done as previously mentioned. Antigenic repair, blockage of endogenous peroxidase, BSA sealing and incubation of primary antibodies were conducted. The residual solution on the slice was discarded, and the secondary antibody labelled HRP was added to the tissue circle, incubated for 60 min at room temperature and away from light, followed by DAB colour development. Haematoxylin staining nucleus ensued, and neutral gum seal piece was performed.

### 2.6 OCs distribution was detected by TRAP staining

Bone decalcification, paraffin embedding section, dewaxing and other experimental operations were the same as previously mentioned. We followed the instructions in the TRAP Staining Kit (Servicebio, China) for the staining process, and images were collected under the microscope. The nuclei of OCs were stained blue with haematoxylin, while their cytoplasm appeared wine‐red. Under a high‐power microscope of 200×, three fields were randomly selected in each section to observe OCs distribution. The Image J software was used to calculate the number of OCs per field.

### 2.7 Immunofluorescence staining

Paraffin sections, dewaxing and rehydration of paraffin sections, antigen repair, blocking endogenous peroxidase, BSA blocking, and primary antibody incubation were performed as previously mentioned. For cell creep, cells were cleaned with PBS and fixed at room temperature for 20–30 min using 4% paraformaldehyde. Further washing with PBS and permeabilizing the film with 0.1%–0.5% Triton X‐100 was conducted for 15–20 min. Cells/Tissues were blocked with 5% BSA and then incubated with primary antibodies Nrp1 (Abcam, The United States), Sema3A (Abcam, The United States), ALP (Servicebio, China) at 4°C overnight. After washing with PBS, fluorescent secondary antibodies were added to the tissue and incubated for 1 h at room temperature while shielding from light. Further washing with PBS and DAPI dye staining for 3–5 min was carried out. After additional washing with PBS, anti‐fluorescence quenching tablet seals were performed.

### 2.8 Isolation and culture of mouse primary DRGs cells

C57BL/6 mice at 4 weeks were sacrificed, and the dorsal root ganglion (DRG) was extracted using micro forceps and micro scissors and placed in αMEM medium. Then DRG was digested in collagenase IV (1 mg/mL) (biofoxx, Germany) for 30 min, centrifuged at 1000 r/min for 5 min, and the supernatant was removed. The DRG was further digested in 0.05% trypsin at 37°C for 30 min. The cells were then cultured for 24–48 h with the medium being changed for the first time and 10 μM cytarabine added to inhibit the proliferation of heterocells. The dorsal root ganglion cells (DRGs) could be seen climbing out of the adherent tissue about 3 days later. Furthermore, the tissue suspension could be directly inoculated into the upper chamber of Transwell with a filtration membrane diameter of 0.8 μm for co‐culture with BMMSCs.

### 2.9 Isolation and stimulation by Sema3A of primary mouse BMMSCs

Six‐week‐old C57BL/6 mice were sacrificed, both femurs and tibia were obtained and immersed in sterile PBS or complete medium. The epiphysis of both ends of the femur was cut with scissors, and the bone marrow cavity contents were washed into a 15 mL centrifugation tube with complete medium. The supernatant was discarded after centrifugation at room temperature for 5 min at 1000 r/min. The cells were resuspended in αMEM complete medium containing 10% foetal bovine serum, 1% penicillin and 1% streptomycin, inoculated in a 10 cm culture dish, and cultured at 37°C and 5% CO_2_ for 24–48 h. The medium was changed every 3 days.

To stimulate BMMSCs, osteogenic induction medium containing 0.5 μg/mL Sema3A (Sino Biological, China) or 0.5 μg/mL Sema3A (Sino Biological, China) + 1 μg/mL SM345431 (MedChemExpress, The United States) was used. The osteogenic induction medium was used in the control group. Gene transcription or protein expression was detected by qRT‐PCR or Western blot 7 days later.

### 2.10 Osteogenic, lipogenic and chondrogenic induced differentiation of BMMSCs

It was operated according to the instructions of the osteogenic, lipogenic and chondrogenic Induction Differentiation Kit (Oricell, China). The induction was terminated after 14–28 days of induction, and Alizarin red staining was performed to observe the formation of calcium nodules. Oil red O staining was done to observe lipid differentiation, and alcian blue staining was performed to observe chondrogenic differentiation.

### 2.11 BMMSCs surface markers were detected by flow cytometry

The BMMSCs cultured in P3 were selected and digested. Cells were counted and transferred into flow tubes with at least 5 × 10^5^ cells per tube. The working liquid was prepared by diluting Fc block with flow buffer at a ratio of 1:200. Then 25 μL working liquid was added into each tube of flow tube, and antibody working solutions CD31 (R&D Systems, The United States), CD29 (R&D Systems, The United States), CD44 (BD Biosciences, The United States) and CD45 (BD Biosciences, The United States) were added to the samples, following the antibody instructions. The samples were centrifuged at room temperature 400 *g* for 5 min and resuspended with 500 μL flow buffer for machine detection.

### 2.12 Osteogenic activity was detected by ALP staining and ALP semiquantitative assay

The BCIP/NBT Alkaline Phosphatase Color Development Kit (Beyotime, China) was used to prepare the BCIP/NBT dyeing solution. The cells were fixed in 4% paraformaldehyde at room temperature for 30 min and then incubated with the BCIP/NBT working solution at 37°C without light for 30 min until the stain depth reached the expected level. The staining solution was discarded, and the cells were washed with PBS. The cells were scanned by a scanner and photographed under a microscope.

For the semiquantitative experiment, cell lysate was collected and centrifuged at 12000 rpm for 10 min at 4°C. The supernatant was transferred to a new EP tube and incubated with ALP sample (reaction substrate) and chromogenic substrate for 5–10 min in the dark at 37°C. The absorbance value was detected at 405 nm wavelength on the enzyme label instrument. Protein concentration was detected by the BCA Protein assay Kit (Thermo Scientific, The United States). ALP activity was calculated by dividing the ALP absorbance value by the total protein content.

To investigate the effect of Sema3A at different concentrations on osteogenic differentiation of BMMSCs, osteogenic induction medium containing 0, 0.1, 0.5 and 1 μg/mL Sema3A (Sino Biological, China) was used, and the medium was changed every 3 days. ALP staining and semiquantitative analysis were performed 7 days later.

### 2.13 Gene transcription were detected by qRT‐PCR

Total RNA was extracted and reverse‐transcribed according to the instructions of the Reverse‐Transcribed kit (Takara, Japan). The samples were prepared per well according to the 10 μL system (cDNA 1 μL, ddH_2_O 3 μL, PCR enzyme 5 μL and primer 1 μL). The 384 well plates of the selected samples were put into a high‐throughput real‐time quantitative fluorescence PCR instrument for amplification, and the formula (2^−ΔΔCT^) was used to analyse the amplification results. *β‐actin* was used as the reference gene, and the amplified osteo‐relative genes included *Alp*, *osteopontin (Opn)* and *Col1*.The specific sequences as follows:

**Table:** 

Gene	Forward primers sequence	Reverse primers sequence
*Alp*	TCCTGCCAAAAACCTCAAAGG	TGCTTCATGCAGAGCCTGC
*Opn*	AGCAAGAAACTCTTCCAAGCAA	GTGAGATTCGTCAGATTCATCCG
*Col1*	TGGGGCAAGACAGTGATCG	GGAGGGAGTTTACAGGAAGCAG
*β‐Actin*	AGGGAAATCGTGCGTGAC	CATACCCAAGAAGGAAGGCT

### 2.14 Proteins expression in BMMSCs were detected by Western blot

The BMMSCs were lysed with Ripa lysis buffer (Beyotime, China), and total protein was prepared and boiled to denature. Subsequently, 20 μg of total protein was loaded into the SDS‐PAGE and run for 2 h. After running the gel, the protein samples were transferred to the PVDF membrane (Millipore, USA), blocked with 5% skim milk, and then incubated with the primary antibodies of GAPDH (Servicebio, China), COL1 (Beyotime, China), OPN (Beyotime, China), RAC1 (Sigma, The United States), p‐RAC1 (Beyotime, China), Nrp1 (Abcam, The United States), Farp1 (Abcam, The United States), Tcf7l1(Sigma, The United States) and Wnt5a (Sigma, The United States) for immunoreaction. The membranes were then washed, incubated with secondary antibodies and visualized using an enhanced chemiluminescence system (Proteinteck, China).

### 2.15 Proliferation capacity of BMMSCs was determined by CCK8 assay

Cells were cultured in αMEM medium containing 0, 0.1, 0.5 and 1 μg/mL Sema3A for 24 or 48 h. Fresh medium was replaced, and 10 μL CCK8 solution (Beyotime, China) was added per well. Cells were then incubated at 37°C and 5% CO_2_ for 2 h. Then the 96‐well plate was put into the Enzyme label detector (TECAN, Austria), and the absorbance was measured at 450 nm.

### 2.16 Bulk RNA‐sequencing and bio‐informatics analysis

BMSCs were cultured in αMEM medium (Invitrogen, USA) supplemented with 0.5 μg/mL Sema3A (Sino Biological, China) or maintained without Sema3A for 3 days. Then total RNA was extracted using Trizol reagent (Invitrogen, USA) and evaluated using the Agilent 2100 Bioanalyzer (Agilent Technologies, USA). RNA integrity number (RIN) ≥ 6.5 were deemed suitable for subsequent analyses. Transcriptome sequencing was conducted on the Illumina sequencing platform (NovaSeq). Transcriptome sequencing libraries were meticulously prepared using the TruSeq‐strand mRNA Library Prep Kit (Illumina, USA). The Pearson correlation coefficient was employed to ascertain the degree of correlation in gene expression patterns among the different samples. Differential gene expression analysis was carried out using the DESeq, and FoldChange ≥1 and adjusted *p* value <0.05 were considered as differentially expressed. The R language Pheatmap software package was harnessed for bidirectional clustering analysis. The STRING database was utilized for protein–protein interaction analysis, and the resultant interaction network was visualized using Cytoscape. Significantly altered genes were subjected to pathway enrichment analysis based on the KEGG database.

### 2.17 siRNA transfection of BMMSCs

To clarify that Sema3A promotes BMMSCs osteogenic differentiation through Wnt pathway, BMMSCs were transfected with the Lipofectamine® 3000 transfection reagent (Thermo Scientific, The United States) according to manufacturer's protocol. BMMSCs were seeded at 3 × 10^5^cells/well on 6‐well plates and cultured in αMEM complete medium containing 10% foetal bovine serum, 1% penicillin and 1% streptomycin. The medium was changed to complete medium containing 0.5 μg/mL Sema3A the next day. BMMSCs were transfected with β‐catenin siRNA or NC siRNA for 24 h, which was repeated on the third day. Cell lysis was harvested at the sixth day. siRNA targeting the β‐catenin gene (Sense: 5′‐GCUGUCCUAUUCCGAAUGUTT‐3′ and antisense: 5′‐ACAUUCGGAAUAGGACAGCTT‐3′) or the negative control siRNA were synthesized by GenePharma Co., Ltd. (Shanghai, China).

### 2.18 Osteogenesis ability of BMMSCs was determined by subcutaneous osteogenesis assay

HA/β‐TCP was immersed in αMEM medium for 24 h. In the experimental group, BMMSCs were cultured in 0.5 μg/mL Sema3A osteogenic induction medium for 7 days and added to HA/β‐TCP, which was then incubated for 24 h. In the control group, BMMSCs were cultured with osteogenic induction medium without Sema3A. The compound was then implanted in the back of the nude mice, and the incisions were closed. After 8 weeks of operation, the nude mice were sacrificed and analysed.

### 2.19 Systematic administration of Sema3A was achieved by intravenous injection

Six‐week‐old C57BL/6 mice received denervation surgery and were injected with Sema3A‐Fc (Sino Biological, China) weekly at a dose of 1 mg/kg for 4 weeks. The control group was injected with PBS buffer solution. The mice were sacrificed 3 days after the last injection, and bilateral tibia were taken for micro‐CT scanning and bone quality parameter analysis.

### 2.20 Statistical analysis

IBM SPSS Statistics 26.0 software was used for statistical analysis. The Shapiro–Wilk normality test was used to test the normality of the variable distribution. The results were expressed as mean ± standard deviation, and the Student *t*‐test or one‐way analysis of variance was used to analyse the differences of the data that exhibited normal distribution. *p* < 0.05 was considered a significant difference.

## 3 RESULTS

### 3.1 Loss of nerve innervation lead to the reduction of bone quality

**FIGURE 1:**
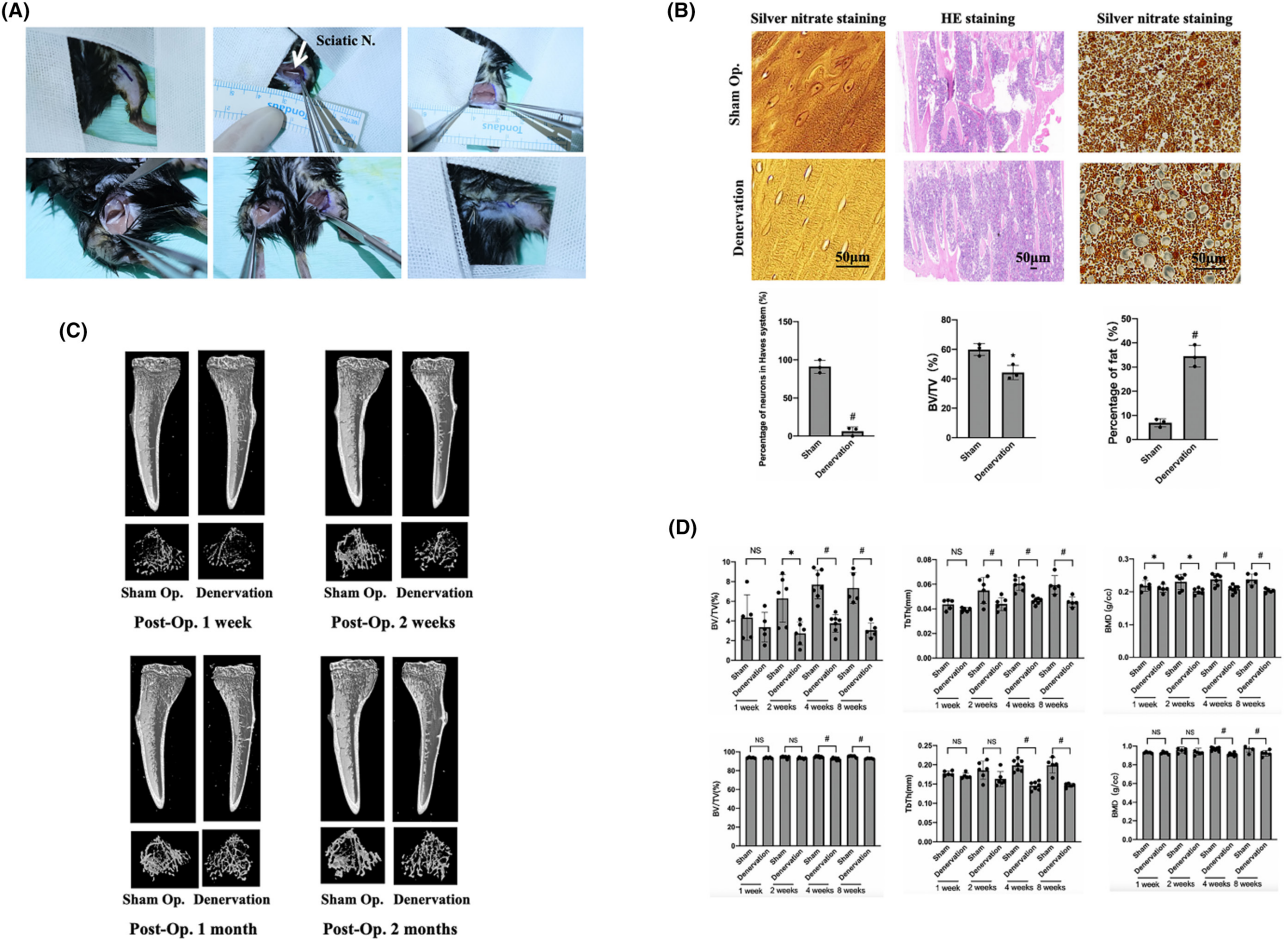
Construction of the mouse tibial denervation model and micro‐CT analysis. (A) Surgery involved severing the sciatic nerve on one side while preserving the other. The arrow indicates the sciatic nerve; (B) Silver nitrate and HE staining of the denervated tibia to detected the nerve fibre distribution, bone trabecular proportion and fat content; (C) micro‐CT scan showing tibia at 1–8 weeks after denervation; (D) Analysis of bone quality parameters. The upper row is the parameters of the cortex and the lower row is the parameters of the cancellous bone. (*n* = 5 for 1 week group and 8 weeks group, *n* = 6 for 2 weeks group, *n* = 7 for 4 weeks group) (Scale: 50 μm). Error bars indicate that the average ± SD Significance levels for statistical analysis are indicated. (*p < 0.05; ^#^ *p* < 0.01). BMD, bone mineral density; BV/TV, bone volume/tissue volume; N., nerve; Op., operation; Tb·Th, tibial trabecular thickness.

To investigate the role of nerve innervation in maintaining bone homeostasis, a mouse tibial denervation model was constructed (Figure 1A). Tibias bones were collected at 1 week, 2 weeks, 4 weeks and 8 weeks after the operation for histological staining and micro‐CT examination. Silver nitrate staining revealed that significantly fewer neurons in the Haversian system of the denervated tibia than in the sham group, indicating that the sciatic nerve severed affecting the peripheral nerve innervation at the same time. The number and thickness of trabecula on the sham side were found to be significantly higher than those on the denervated side. Additionally, the fat content in the denervated tibia was significantly higher than that of the sham operation group (Figure 1B), suggesting that the decrease in tibia mass after the loss of innervation may be related to the ageing of BMMSCs and their differentiation towards adipogenesis. micro‐CT revealed that the tibial trabecula on the denervated side were less numerous and thinner than those on the sham side from the first week after surgery (Figure 1C), and this difference became more pronounced over time. Statistical analysis of bone quality related parameters showed that the bone mineral density (BMD) on the denervated side was significantly lower than that on the sham side 1 week after surgery. At 2 weeks after surgery, bone volume/tissue volume (BV/TV) and tibial trabecular thickness (Tb·Th) on the sham operation side were significantly higher than those on the denervation side. Cortical bone began to exhibit significant differences 4 weeks after surgery, which was later but consistent with the trend of cancellous bone. This indicates that bone homeostasis can be affected by denervation, resulting in decreased bone mass, and that cancellous bone changes earlier than cortical bone (Figure 1D).

### 3.2 Sensory nerves regulate the activity of BMMSCs through neurotransmitters

**FIGURE 2:**
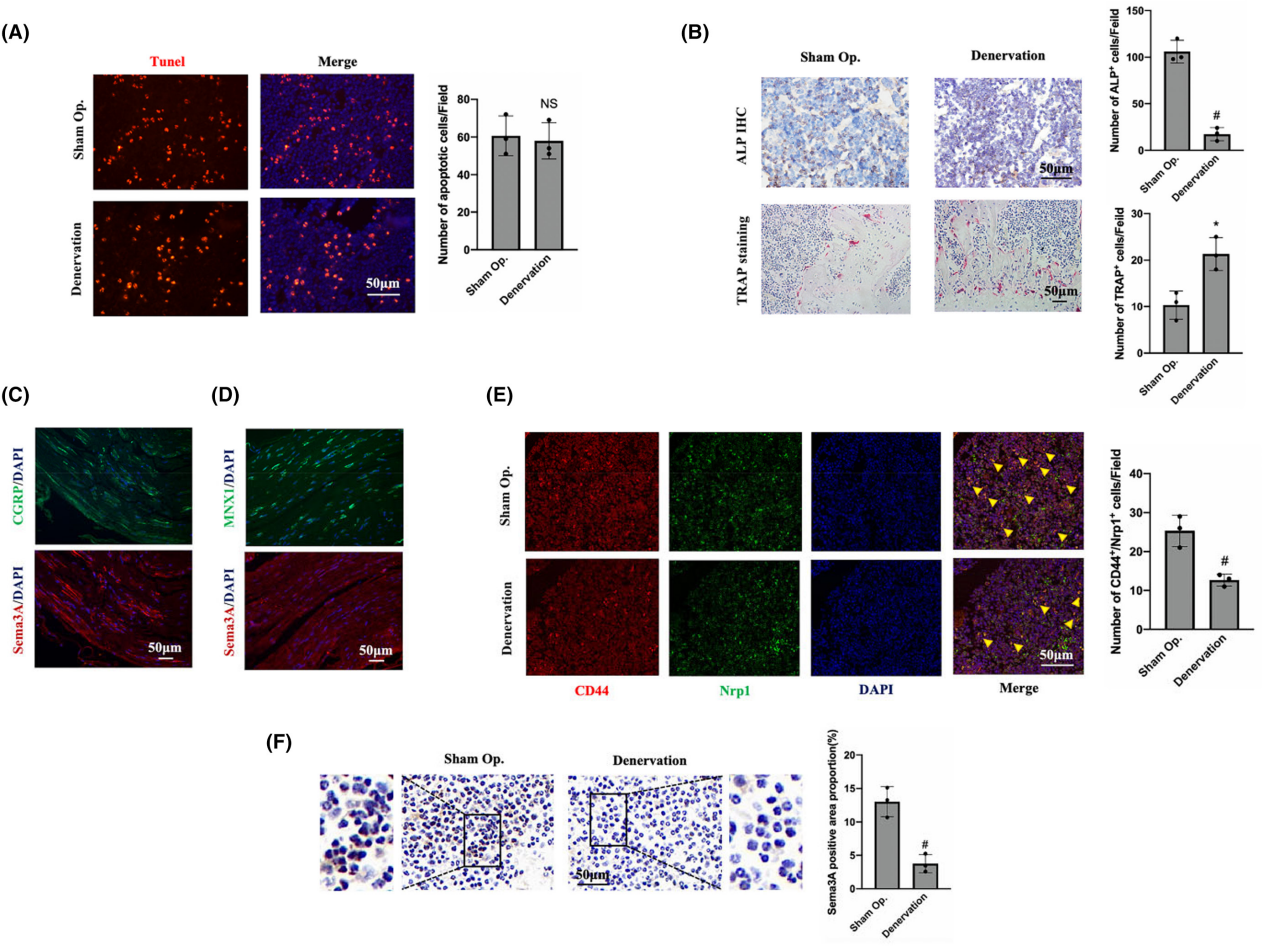
Sensory nerves regulate the number and activity of BMMSCs through the secretion of neurotransmitters, thus regulating bone homeostasis. (A) Tunel staining of denervated tibia; (B) ALP immunohistochemical staining and TRAP staining of denervated tibia; (C) CGRP and Sema3A immunofluorescence staining in successive sections of the sciatic nerve; (D) MNX1 and Sema3Aimmunofluorescence staining in successive sections of the sciatic nerve; (E) CD44 and Nrp1 immunofluorescence costaining of tibia. Yellow arrows indicate cells coexpressing CD44 and Nrp1; (F) Sema3A immunohistochemical staining of denervated tibia. (Scale: 50 μm). Error bars indicate that the average ± SD. Significance levels for statistical analysis are indicated. (*p < 0.05; ^#^ *p* < 0.01). Op., operation.

Tunel staining was performed to determine whether changes in bone homeostasis after denervation were related to cell apoptosis. The results showed that the number of apoptotic cells in the two groups were similar (Figure 2A), which initially excluded the changes in bone homeostasis caused by cell apoptosis. Immunohistochemical staining indicated that the number of cells with positive ALP expression was 110.89 ± 22.03 in the sham operation group, which was significantly higher than 18.13 ± 7.92 in the denervation group (Figure 2B), suggesting decreased osteogenic activity of the denervated tibia. However, the number of OCs in the denervated tibia were 22.07 ± 8.86, significantly higher than that in the sham side (9.98 ± 5.84; Figure 2B), indicating increased osteoclast activity. In conclusion, histological staining and analysis of the denervated tibia indicated that loss of innervation results in ageing of BMMSCs and differentiation into adipocytes rather than OBs, with an increase of OCs and a decline in bone quality.

Since the sciatic nerve is a mixed nerve, comparative analysis was conducted through continuous section and immunofluorescence staining to clarify the relationship between Sema3A and sensory or motor neurons. CGRP is often considered as a marker of sensory nerves, while MNX1 is frequently used as a marker of motor neurons. Immunofluorescence staining showed that Sema3A and CGRP were expressed in similar locations, that is, Sema3A was simultaneously expressed in CGRP‐positive neurons (Figure 2C). However, MNX1‐positive motor neurons did not express Sema3A (Figure 2D). These results suggest that Sema3A is expressed in the sensory neurons of the sciatic nerve rather than in the motor neurons. Through CD44 and Nrp1 immunofluorescence costaining, it was found that the number of Nrp1‐positive BMMSCs after denervation was significantly lower than that on the sham operation side (Figure 2E). Importantly, Sema3A expression in the tibia of denervation side was significantly lower than that in the sham side (Figure 2F). This suggests that the imbalance of bone homeostasis may be related to the decrease in the number of BMMSCs, and the Sema3A‐Nrp1 signalling may play an important role.

### 3.3 DRGs can promote osteogenic differentiation of BMMSCs

DRGs were isolated and cultured from the dorsal root ganglion tissue of 4‐week‐old B57CL/6 mice using the tissue adhesion method. The DRGs' neuron cell bodies and elongated nerve fibres were visible under light microscopy (Figure S1A). Sema3A expression was observed in the cytoplasm of the dorsal root ganglia by immunofluorescence staining (Figure S1A), consistent with staining results of sensory neurons expressing Sema3A in the sciatic nerve of mice. BMMSCs were isolated and cultured using the whole bone marrow adherent method, showing primary colony growth. After 21–28 days of osteogenic, adipogenic and chondrogenic induction culture, the cells were stained. Calcium nodules were visible during osteogenesis, round lipid droplets were visible during adipogenesis, and endo‐acid mucopolysaccharides in chondrospheres were stained blue after chondrogenesis. It was confirmed that the isolated and cultured BMMSCs had the potential to differentiate into osteogenesis, lipogenesis and chondrogenesis (Figure S1B). BMMSCs of P3 were selected and identified by flow cytometry. Approximately 99.97% of the cells expressed stem cell markers CD29 and CD44, while only 0.21% of the cells expressed vascular endothelial cell surface markers CD31 and 0.07% expressed leukocyte common antigen CD45 (Figure S1C). Therefore, the cultured cells isolated from mouse bone marrow can be considered as BMMSCs. Additionally, immunofluorescence staining showed that BMMSCs expressed Nrp1, the specific receptor of Sema3A (Figure S1D), thus providing a basis for exploring the effect and pathway of Sema3A on the osteogenic activity of BMMSCs.

**FIGURE 3:**
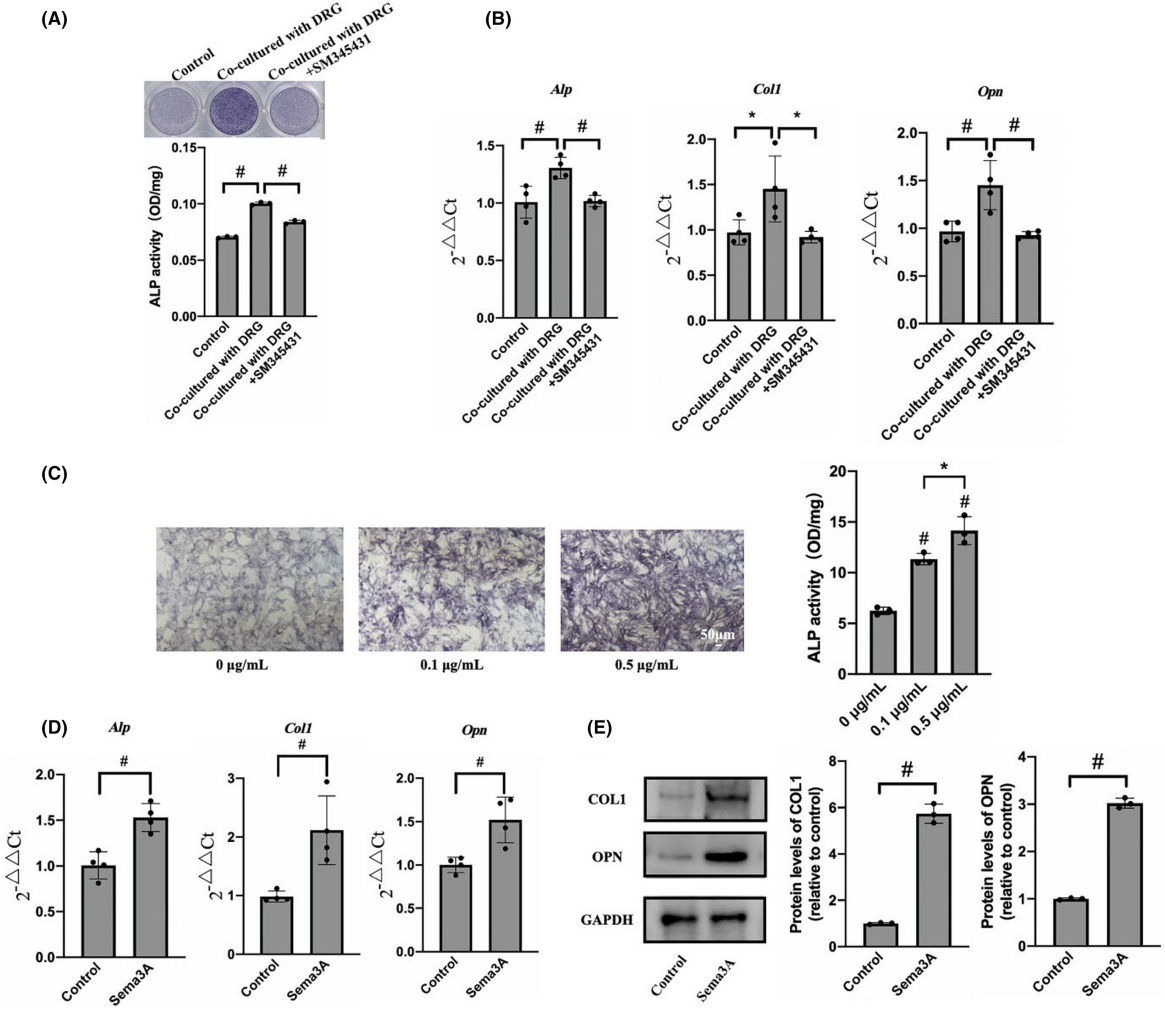
Cocultured with DRGs or stimulated by sema3A promote osteogenic differentiation of BMMSCs. (A) ALP staining and semiquantitative results of BMMSCs co‐cultured with DRGs in a Transwell chamber for 7 days; (B) Transcription levels of *Alp*, *Col1* and *Opn* were detected by qRT‐PCR of BMMSCs co‐cultured with DRGs through a Transwell chamber for 7 days. (C) ALP staining and semiquantification of BMMSCs cultured in osteogenic induction solution containing different concentrations of Sema3A for 7 days. (D) Expression levels of *Alp*, *Col1* and *Opn* detected by qRT‐PCR. (E) Expression levels of COL1 and OPN detected by Western blot. (Scale: 50 μm). Error bars indicate that the average ± SD. Significance levels for statistical analysis are indicated. (*p < 0.05; ^#^ *p* < 0.01).

To investigate the interaction between BMMSCs and DRGs, BMMSCs were inoculated in the lower compartment of Transwell, while DRGs were inoculated in the upper compartment for co‐culture. ALP staining and semiquantitative detection results showed that the activity of ALP in the co‐culture group was 0.10 ± 0.001, which was significantly higher than that in the control group 0.07 ± 0.0007 (Figure 3A). These results indicated that co‐culture with DRGs could significantly enhance the osteogenic activity of BMMSCs. The addition of Sema3A inhibitor SM345431 significantly inhibited the increase in osteogenic activity of BMMSCs caused by co‐culture with DRGs (Figure 3A). To further clarify the effect of co‐culture with DRGs on the expression of osteogenic genes of BMMSCs, qRT‐PCR was performed. The transcription levels of *Alp* and *Col1* in the DRGs co‐culture group were significantly higher than those in the control group. Furthermore, the transcription level of *Opn* in the co‐culture group was 1.55 ± 0.22 times than that in the control group (Figure 3B). Therefore, co‐culture with DRGs can increase the transcription of osteogenic genes such as *Alp*, *Opn* and *Col1*, promoting osteogenic differentiation of BMMSCs. The transcription of *Alp*, *Opn* and *Col1* in BMMSCs was significantly inhibited after adding Sema3A inhibitor SM345431 (Figure 3B). It suggests that the enhancement of osteogenic differentiation of BMMSCs was closely related to Sema3A secreted by DRGs.

### 3.4 Sema3A maintain bone homeostasis by promoting osteogenic of BMMSCs

To explore the effects of Sema3A on osteogenic differentiation and proliferation of BMMSCs, the cells were cultured with osteogenic induction solution containing 0, 0.1 and 0.5 μg/mL recombinant factor Sema3A for 7 days. The ALP activity of the BMMSCs in the presence of 0.1 μg/mL and 0.5 μg/mL Sema3A were significantly enhanced compared to the control group (Figure 3C). Similar results were obtained from ALP staining, demonstrating that Sema3A significantly promoted the osteogenic activity of BMMSCs. Furthermore, the stimulation of BMMSCs with 0.5 μg/mL Sema3A for 7 days showed an increase in the expression levels of *Alp*, *Col1* and *Opn* by 1.53 ± 0.15 times, 2.12 ± 0.72 times and 1.52 ± 0.26 times, respectively, compared to the control group (Figure 3D). Based on the qRT‐PCR results, COL1 and OPN were chosen as target proteins representing osteogenic activity. Following the Sema3A stimulation, the expression levels of COL1 and OPN in BMMSCs were 5.73 ± 0.34 times and 3.02 ± 0.09 times higher than those in the control group, respectively (Figure 3E). In addition, the effect of Sema3A on the proliferation of BMMSCs was evaluated by CCK8 assay. The results showed no significant difference between the groups following the exposure to 0.1, 0.5 or 1 μg/mL Sema3A for 24 or 48 h (Figure S1E,F). Hence, Sema3A could not promote the proliferation of BMMSCs. In summary, our findings indicate that Sema3A can maintain bone homeostasis by promoting the osteogenic differentiation of BMMSCs, without affecting their proliferation.

### 3.5 Sema3A promoted osteogenic differentiation of BMMSCs through a Wnt/β‐catenin/Nrp1 positive feedback loop

**FIGURE 4:**
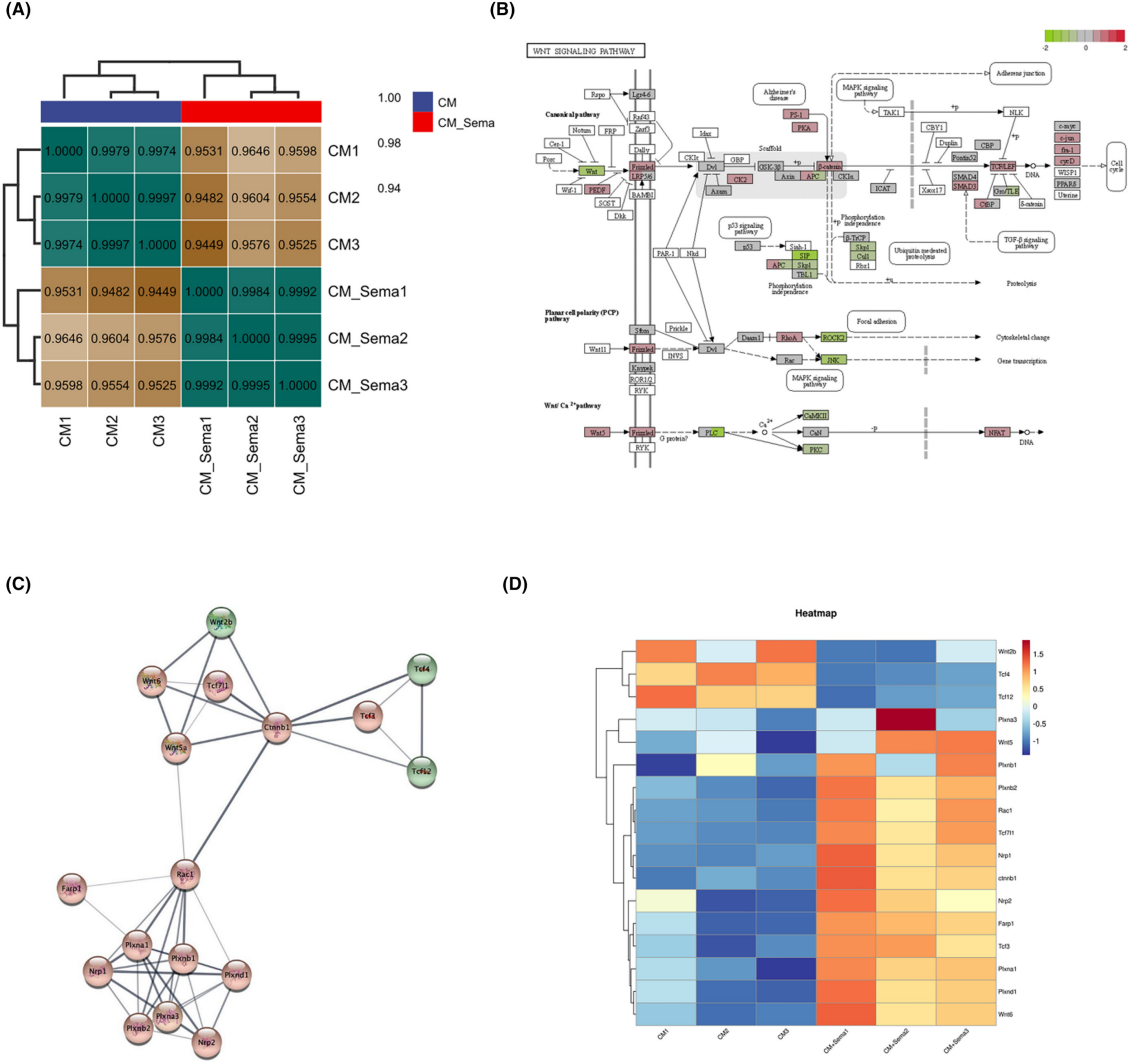
Sema3A affects Wnt signalling in BMMSCs. (A) Correlation analysis of gene expression between CM group and Sema3A stimulated group; (B) Signal map of the Wnt pathway. red: upregulated genes and green: downregulated genes; (C) The protein–protein interaction network analysis of the differentially expressed genes between CM group and Sema3A stimulated group. Sema3A receptor and Wnt pathway were highly expressed; (D) Cluster analysis of key changed genes related to the receptor of Sema3A and Wnt pathway between CM group and Sema3A stimulated group. Heat map showing the Z‐score of selected gene expression.

To explore the specific mechanism by which Sema3A affects BMMSC, we performed transcriptome sequencing and bioinformatics analysis. BMMSC were divided into complete medium (CM) group and Sema3A stimulation group (CM + Sema3A) with three samples in each group. BMMSC were cultured in complete medium (αMEM+10% FBS+ 1% Pen/Strep) supplemented with 0.5 μg/mL Sema3A in the CM + Sema3A group, while those in the CM group were cultured without SEMA3A. Total RNA was extracted 3 days later for transcriptome sequencing and bioinformatics analysis. The results of correlation analysis showed that the expression patterns of samples from CM group and Sema3A group were relatively similar, and the samples had good biological duplication (Figure 4A). We observed that the Wnt signalling was strongly activated at the transcriptional level through the Kyoto Encyclopedia of Genes and Genomes (KEGG) enrichment analysis (Figure 4B). Importantly, the transcriptional level changes and interaction modules of Sema3A receptors Nrp1, Plxna1 and Wnt pathway related proteins, such as Rac1, β‐catenin and Wnt6 were enriched by protein–protein interaction analysis (Figure 4C). Heat maps showed a unique cluster of gene expression between the CM and Sema3A samples (Figure 4D).

**FIGURE 5:**
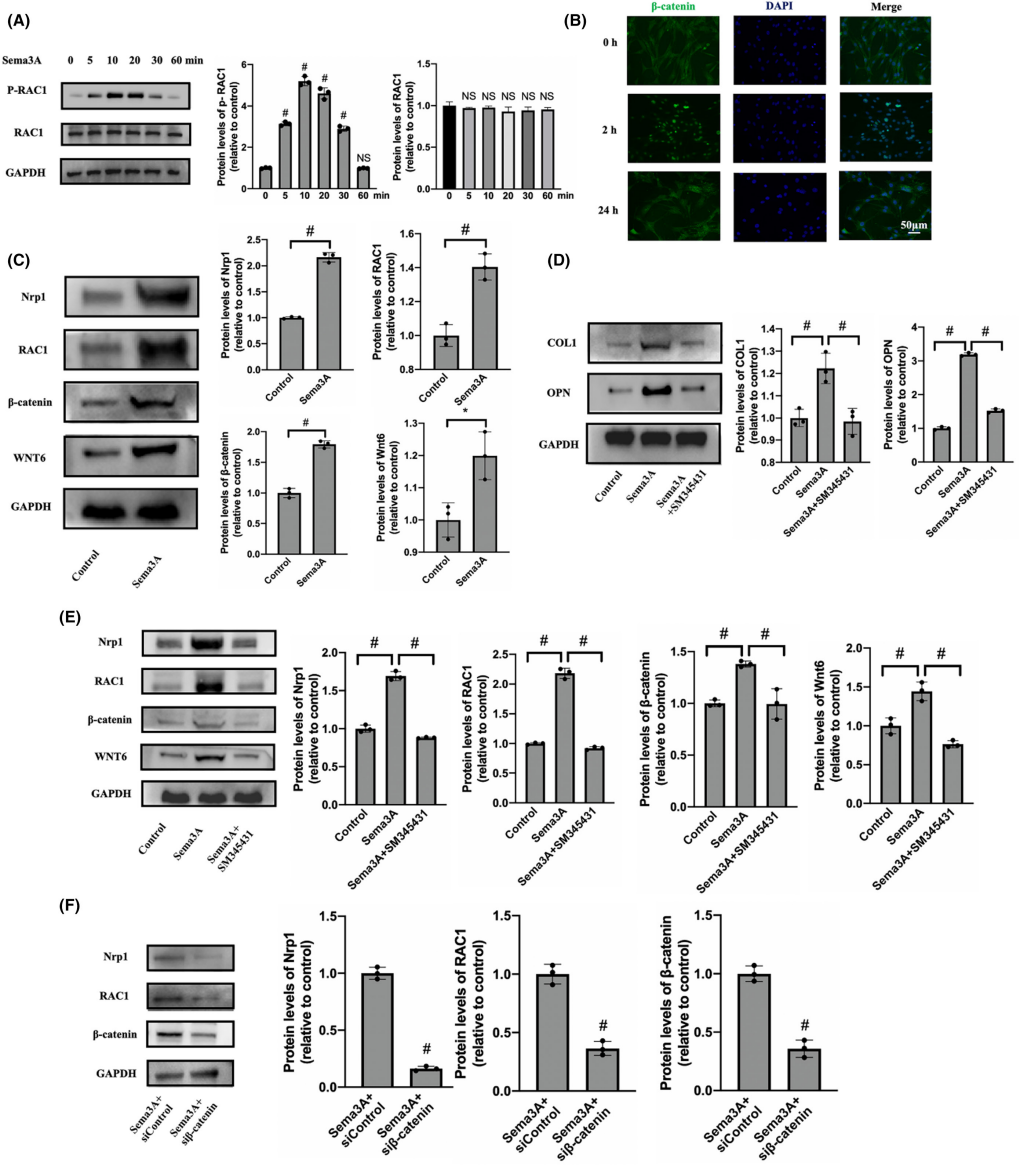
Sema3A promotes osteogenic differentiation of BMMSCs through the Wnt/β‐catenin/Nrp1 positive feedback loop. (A) Expression levels of RAC1 and p‐RAC1 determined by Western blot after stimulation with Sema3A; (B) Localization of β‐catenin detected by immunofluorescence staining. After 2 h of Sema3A stimulation of BMMSC, significant cytoplasmic to nuclear transfer was observed; (C) Expression levels of WNT pathway‐related proteins and Nrp1 determined by Western blot; (D) Effects of Sema3A and its inhibitor SM345431 on the expression levels of COL1 and OPN analysed by Western blot; (E) and (F) Expression levels of Wnt pathway‐related proteins and Nrp1 determined by Western blot. (Scale: 50 μm). Error bars indicate that the average ± SD. Significance levels for statistical analysis are indicated. (*p < 0.05; ^#^ *p* < 0.01; NS, no significance).

The Wnt/β‐catenin signalling pathway is crucial for maintaining bone homeostasis as it recruits BMMSCs and differentiates them into OBs. Stimulation of BMMSCs with 0.5 μg/mL Sema3A led to a significant increase in RAC1 phosphorylation levels over time, with the highest level being reached around 10 min (Figure 5A). Meanwhile, β‐catenin significantly transferred and accumulated in the nucleus 2 h after stimulation (Figure 5B). Our results suggest that Sema3A binds to Nrp1 and activates the classical Wnt/β‐catenin signalling pathway, promoting RAC1 phosphorylation and β‐catenin transfer into the nucleus, thus promoting osteogenic differentiation of BMMSCs. In addition, the expressions of Rac1, β‐catenin and WNT6 in BMMSCs were significantly increased compared to the control group (Figure 5C). Notably, Nrp1 expression was also increased by 2.16 ± 0.07 times, indicating that Sema3A might promote Nrp1 expression by activating the classical Wnt/β‐catenin signalling pathway, thereby positively regulating Wnt signalling and osteogenic differentiation in BMMSCs.

SM345431, a Sema3A‐specific inhibitor, inhibits the binding of Sema3A to Nrp1. When SM345431 was added with Sema3A, the expression levels of COL1 and OPN were significantly decreased (Figure 5D). Additionally, Western blot revealed that SM345431 or β‐catenin siRNA both significantly inhibited the expression levels of Nrp1, RAC1, β‐catenin and WNT6 compared to the Sema3A stimulation group (Figure 5E,F). In summary, our findings show that SM345431 effectively inhibits the osteogenic differentiation induced by Sema3A, as well as inhibit the Wnt/β‐catenin pathway. Importantly, Nrp1 expression is significantly inhibited during this process.

### 3.6 Sema3A promotes osteogenic differentiation by maintaining the vitality of BMMSCs, leading to improved bone quality in vivo

**FIGURE 6:**
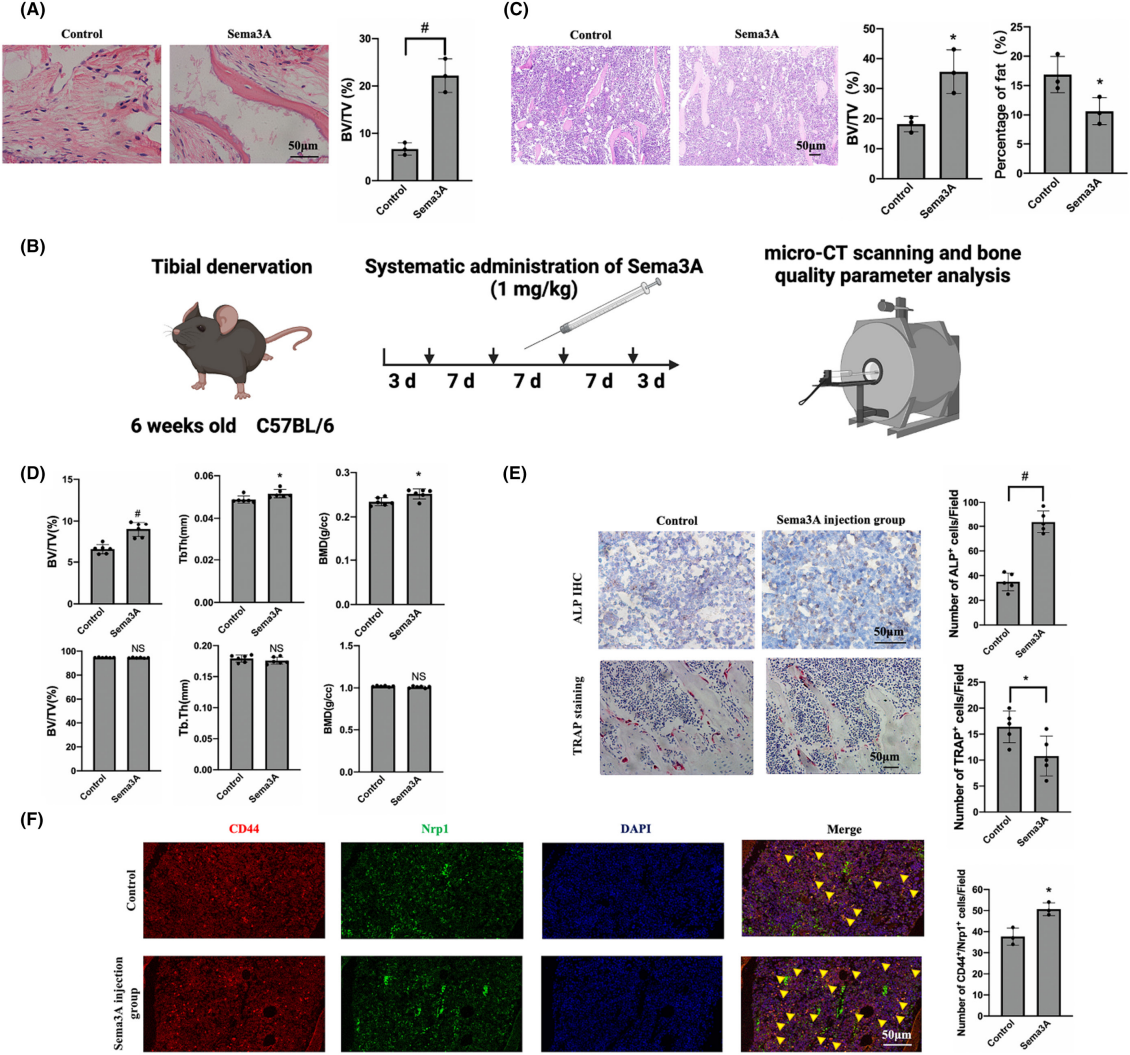
Sema3A promotes osteogenesis and improves bone quality by maintaining the vitality of BMMSCs in vivo. (A) Subcutaneous osteogenic effect of BMMSCs stimulated by Sema3A in nude mice determined by HE staining; (B) The schematic representation showing administration of Sema3A treatment to rescue the phenotype of tibial denervation surgery; (C) BV/TV and fat proportion analysed by HE staining after systematic administration of Sema3A by intravenous injection; (D) Analysis of parameters related to bone quality (*n* = 6 in each group); (E) Osteogenic and osteoclastic activity determined by ALP immunohistochemical staining and TRAP staining; (F) Immunofluorescence costaining analysis of the cell distribution of CD44 and Nrp1 coexpression in the bone marrow. Yellow arrows indicate cells coexpressing CD44 and Nrp1. (Scale: 50 μm). Error bars indicate that the average ± SD, and all experiments are tripled. Significance levels for statistical analysis are indicated. (*p < 0.05; ^#^ *p* < 0.01).

Subcutaneous osteogenesis of BMMSCs treated with Sema3A showed significantly higher bone volume ratio when compared to the control group (Figure 6A). Then we try to rescue the phenotype of tibial denervation surgery by administering Sema3A treatment (Figure 6B). Systemic administration of Sema3A increased trabecular volume ratio to (35.66 ± 5.94) % as compared to the (18.22 ± 2.11) % in control group (Figure 6C), whereas bone volume ratio, trabecular thickness and bone density were significantly increased in Sema3A treated group by 1.36 ± 0.12 times, 1.06 ± 0.04 times and 1.07 ± 0.04 times, respectively, compared to the control group (Figure S2A). However, there was no significant difference in cortical bone density between the two groups (Figure 6D). Furthermore, administration of Sema3A increased the number of ALP‐positive cells, and significantly decreased the number of TRAP‐positive cells (Figure 6E). Notably, the number of BMMSCs coexpressed by CD44 and Nrp1 was significantly higher in the Sema3A injection group than in the control group (Figure 6F). Systemic use of Sema3A had no significant effect on inhibiting apoptosis of bone marrow cells (Figure S2B).

**FIGURE 7:**
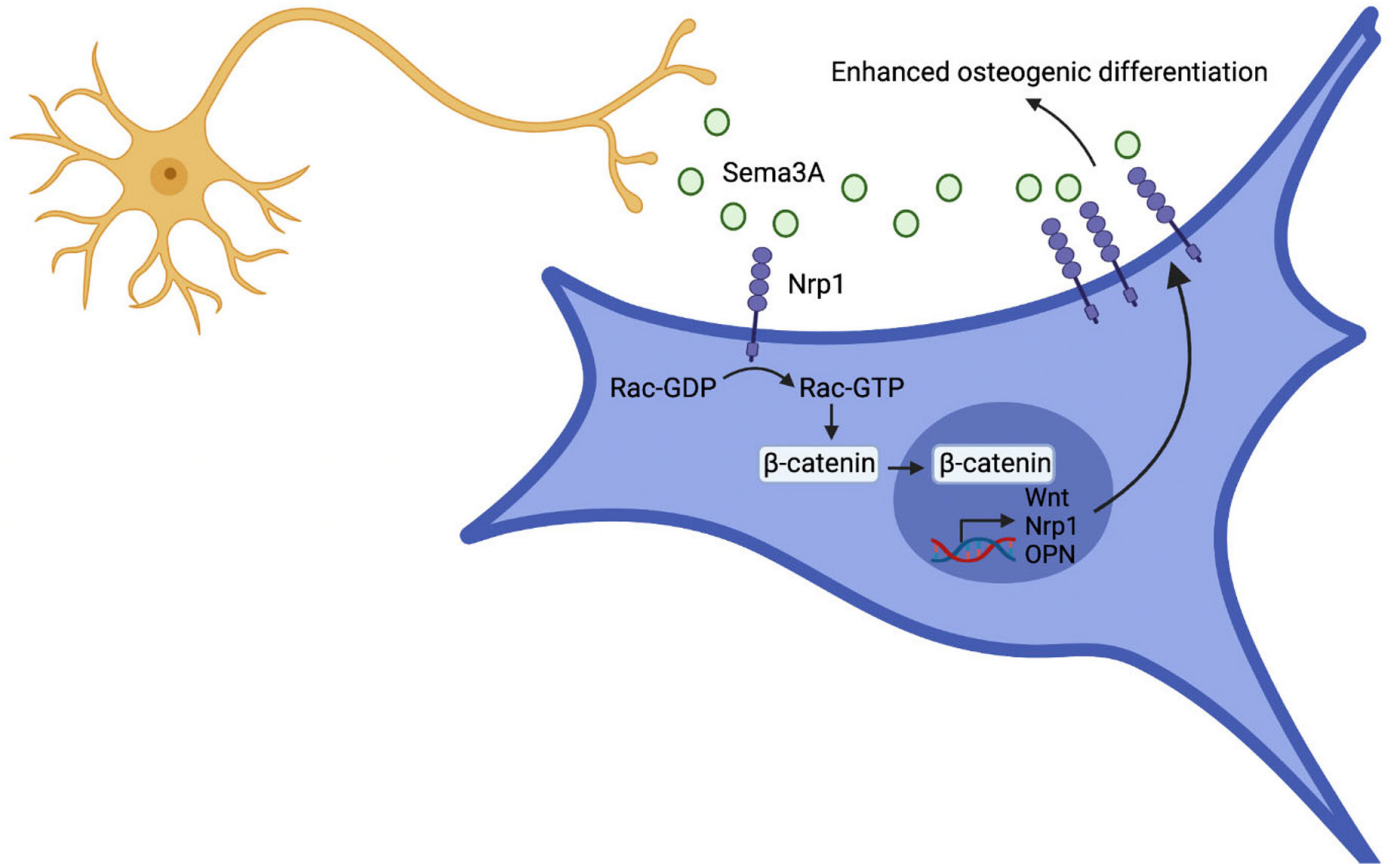
Schematic diagram of Sema3A promoting osteogenic differentiation of BMMSCs by activating the Wnt/β‐catenin/Nrp1 positive feedback loop. After co‐culture with DRGs or stimulation by Sema3A, transcription and protein expression of nrp1 in BMMSCs are promoted by activating classical Wnt/β‐catenin signalling, forming a positive feedback loop that amplifies the effect of Sema3A and promotes osteogenic differentiation of BMMSCs.

In summary, systemic administration of Sema3A significantly improved cancellous bone quality, reduced fat content and promoted bone growth. Moreover, there were no observed pathological changes or abnormal behaviour in vital organs after intravenous administration of Sema3A. Our results suggest that binding of Sema3A to Nrp1 can enhance the effect of Sema3A on BMMSCs through the positive feedback loop, regulating the transcription of *nrp1* and other genes via WNT pathway, leading to increased expression of Nrp1 and promoting the enhancement of BMMSC activity (Figure 7). Hence, Sema3A can be a promising drug for the treatment of bone degradation‐related diseases.

## 4 DISCUSSION

Bone metabolism remains stable when osteogenic activity is balanced with bone resorption and formation activity. A study showed that innervation could promote bone remodelling during tooth movement in the rabbit model of inferior alveolar nerve injury.^26^ In the present study, sciatic nerve injury caused a decrease in tibia bone quality and an increase in adipose tissue content of cancellous bone. Immunohistochemical staining indicated that changes in bone homeostasis were associated with decreased osteoblast activity and increased osteoclast activity, but not with cell apoptosis. Notably, the number of BMMSCs coexpressed by CD44 and Nrp1 in denervated tibia significantly decreased, indicating that the imbalance of bone homeostasis may be caused by the bidirectionalism of adipogenic differentiation of BMMSCs and the enhancement of osteoclast activity.

Nrp1 forms a receptor complex with PlxnA1, with PlxnA1 containing a near‐membrane sequence that includes three basic amino acid residues (KRK), necessary for FARP2 binding. Effective recruitment of FARP2 requires the extracellular domain of Nrp1.^27^ Upon binding to the Nrp1‐PlxnA1 complex receptor, Sema3A promotes FARP2 expression within 10 min.^21^, ^28^, ^29^ Sema3A also induces FARP2 separation from PlxnA1, activating Racl, which promotes the nuclear accumulation of β‐catenin.^17^, ^30^ Interestingly, Sema3A knockout mice exhibit not only reduced bone formation and increased bone degradation but also inhibited expression of Rac1 and FARP proteins involved in the Wnt signalling pathway.^24^ Sema3A plays a bone protective role through the activation of the classical Wnt/β‐catenin pathway, although its specific mechanisms remain unclear. This study focused on the positive feedback loop formed between BMMSCs co‐cultured with DRGs or stimulated by Sema3A, promoting Nrp1 expression by activating the classical Wnt/β‐catenin signalling. This loop amplifies the effect of Sema3A and promotes BMMSC differentiation into OBs.

Most hormone or drug therapies used for bone degeneration simultaneously decrease bone formation. For instance, parathyroid hormone injections can increase bone production but can also promote bone degradation. Bisphosphonates effectively slow bone resorption, but their limitation is that they also reduce bone formation. To determine whether Sema3A presents a feasible clinical treatment option for degenerative bone diseases, a study by Hayashi et al^24^ involved injecting of Sema3A into wild‐type mice, resulting in increased trabecular bone volume, trabecular bone parameters and the number of bone cells, with a concurrent decrease in osteoclasts. Similarly, intravenous injection of Sema3A has been shown to promote new bone formation in a mouse bone defect model.^24^ Our study also demonstrated that systemic application of Sema3A promotes BMMSC activity while suppressing OCs activity, resulting in a dual bone protective effect. These findings provide a basis for the prevention of osteoporosis and the development of more biomimetic tissue‐engineered bone. Taken together, Sema3A displays promising potential as a therapeutic agent for treating bone degenerative diseases.

### 4.1 Significance statement

1. This study explores the mechanism of Sema3A‐mediated regulation of osteogenic differentiation of BMMSCs, through the Nrp1/WNT positive feedback loop.
2. Provide a solid and systematic experimental basis for understanding the neuroregulation of bone remodelling and for the development of biomimetic tissue‐engineered bone.

### 4.2 Limitations of the study

Some limitations of the current study need to be acknowledged. Firstly, to clarify the role of Sema3A in regulating BMMSCs activity through Nrp1/WNT positive feedback loop, it is necessary to downregulate the WNT pathway using siRNA. Besides, the bone quality of the adult Sema3A knockout (KO) mice can be examined using micro‐CT and histological analysis, and whether the osteoporotic phenotype can be rescued by Sema3A need to be explored. Additionally, molecular experiments such as qRT‐PCR and Western Blot can be conducted to examine the expression of Nrp1, Rac1, β‐catenin in Sema3A KO mouse. These experiments will provide further evidence that sensory nerves maintain bone homeostasis via the Sema3A/Nrp1/WNT positive feedback loop.

## AUTHOR CONTRIBUTIONS

**Jingcun Shi:** Conceptualization (equal); data curation (equal); formal analysis (equal); writing – original draft (lead); writing – review and editing (equal). **Bingqing Zhang:** Data curation (equal); writing – review and editing (lead). **Ziqian Wu:** Writing – review and editing (equal). **Yuhan Zhang:** Writing – review and editing (equal). **Anand Gupta:** Writing – review and editing (equal). **Xudong Wang:** Writing – review and editing (equal). **Jieyu Wang:** Writing – review and editing (equal). **Lisha Pan:** Writing – review and editing (equal). **Meng Xiao:** Writing – review and editing (equal). **Shijian Zhang:** Writing – review and editing (equal). **Lei Wang:** Conceptualization (lead); funding acquisition (supporting); project administration (lead); writing – review and editing (equal).

## FUNDING INFORMATION

This study was supported by the grants from National Natural Science Foundation of China (No. 81970907 and No.81771046) and Shanghai Municipal Health Commission (No. 202340135).

## CONFLICT OF INTEREST STATEMENT

The authors declare no competing interests.

## Supporting information


Figures S1–S2.

## Data Availability

The datasets supporting the conclusions of this article are included within the article.
