# Supplementary material for: Peripheral nerve‐derived Sema3A promotes osteogenic differentiation of mesenchymal stem cells through the Wnt/β‐catenin/Nrp1 positive feedback loop
Source: J Cell Mol Med. 2024 Apr 3;28(8):e18201. doi: 10.1111/jcmm.18201 (PMC10989576; doi:10.1111/jcmm.18201)
Supplement: Supplementary file 1 — Figures S1–S2. [file JCMM-28-e18201-s001.docx]

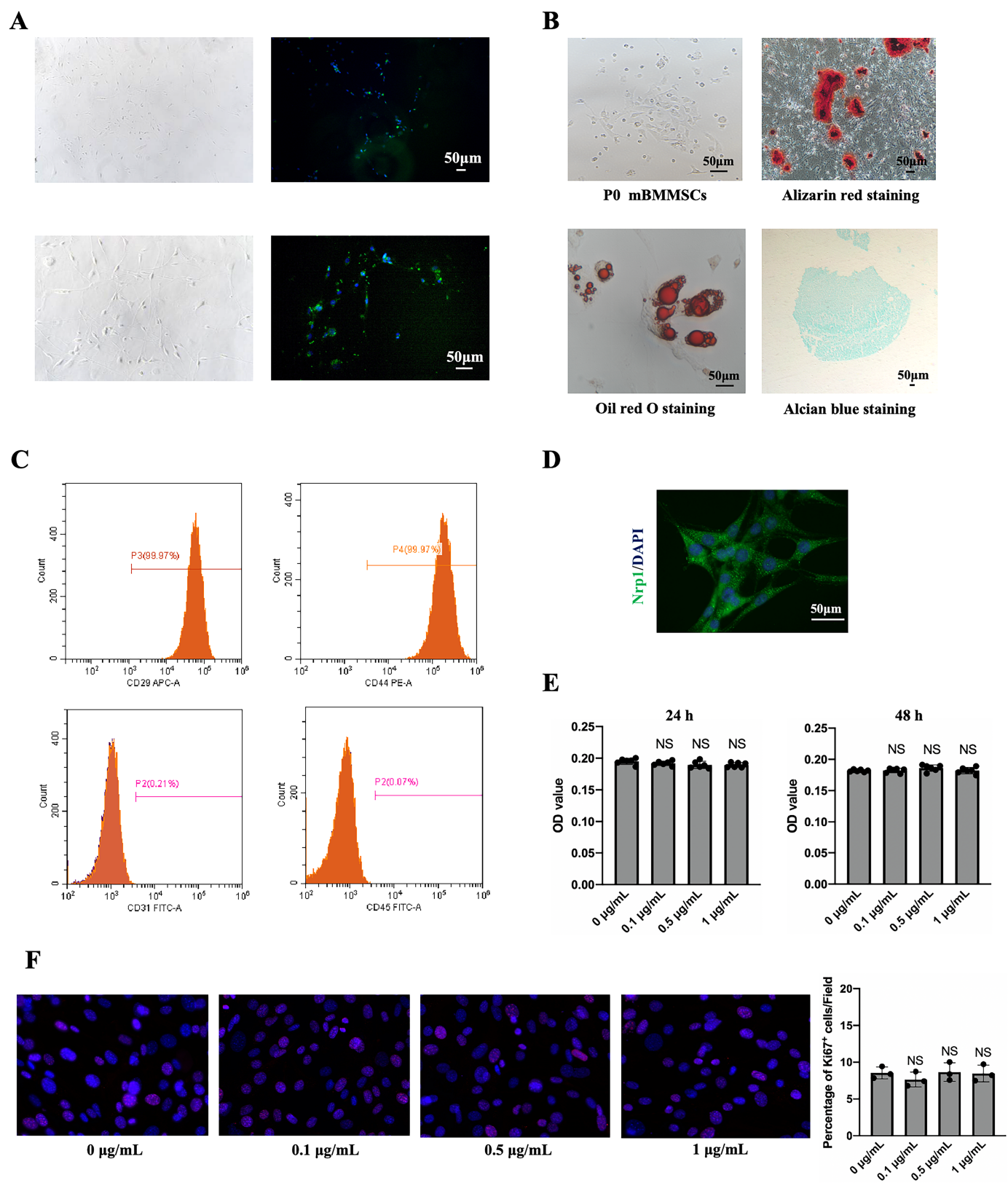


**Supplementary Figure 1** DRGs and BMMSCs express receptors and ligands that bind to each other

(A) Morphology and Sema3A immunofluorescence staining of primary DRGs; (B) Primary BMMSCs grew into colonies, alizarin red, oil red O, and alcian blue staining performed after osteogenesis, lipogenesis, and chondrogenesis, respectively; (C) BMMSC surface markers were identified by flow cytometry; (D) Nrp1 expression in BMMSCs was determined by immunofluorescence staining; (E) Effects of Sema3A on the proliferation of BMMSCs detected by CCK8; (F) The proliferation capacity of SEMA3A-stimulated BMMSCs was characterized by Ki67 immunofluorescence staining. (Scale: 50 μm). Error bars indicate that the average ± SD. Significance levels for statistical analysis are indicated. (NS, no significance).


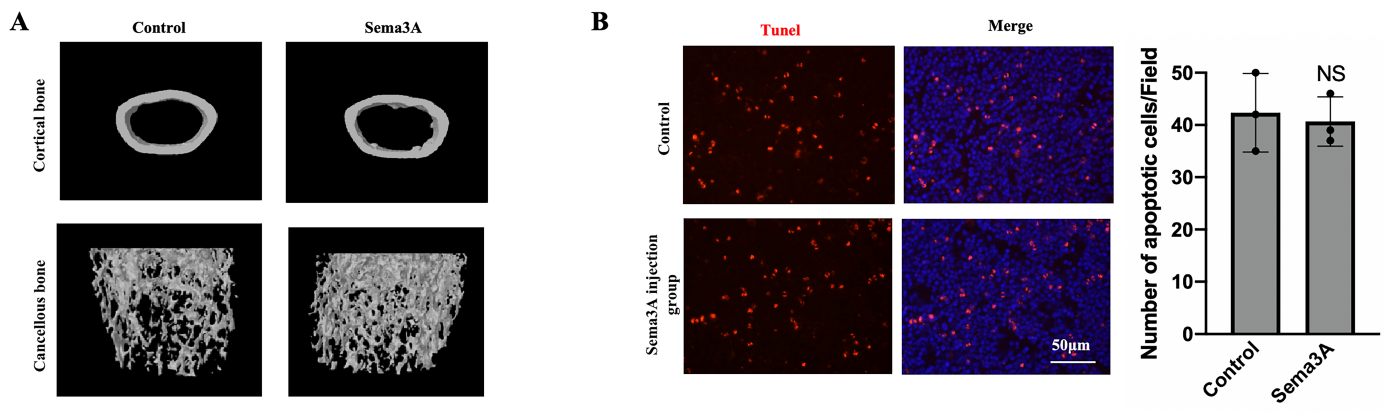


**Supplementary Figure 2** Imaging and apoptosis levels were measured after systematic administration of Sema3A

(A) Bone morphology detected by micro-CT. (B) Cell apoptosis analyzed by TUNEL staining. (Scale: 50 μm).
